# Next generation sequencing based in-house HIV genotyping method: validation report

**DOI:** 10.1186/s12981-021-00390-8

**Published:** 2021-10-02

**Authors:** Alisen Ayitewala, Isaac Ssewanyana, Charles Kiyaga

**Affiliations:** National Health Laboratories and Diagnostic Services, Central Public Health Laboratories, Ministry of Health, P.O Box 7272, Kampala, Uganda

**Keywords:** HIV Genotyping, Next Generation Sequencing, Sanger Sequencing, Uganda

## Abstract

**Background:**

HIV genotyping has had a significant impact on the care and treatment of HIV/AIDS. At a clinical level, the test guides physicians on the choice of treatment regimens. At the surveillance level, it informs policy on consolidated treatment guidelines and microbial resistance control strategies. Until recently, the conventional test has utilized the Sanger sequencing (SS) method. Unlike Next Generation Sequencing (NGS), SS is limited by low data throughput and the inability of detecting low abundant drug-resistant variants. NGS can improve sensitivity and quantitatively identify low-abundance variants; in addition, it has the potential to improve efficiency as well as lowering costs when samples are batched. Despite the NGS benefits, its utilization in clinical drug resistance profiling is faced with mixed reactions. These are largely based on a lack of a consensus regarding the quality control strategy. Nonetheless, transitional views suggest validating the method against the gold-standard SS. Therefore, we present a validation report of an NGS-based in-house HIV genotyping method against the SS method in Uganda.

**Results:**

Since there were no established proficiency test panels for NGS-based HIV genotyping, 15 clinical plasma samples for routine care were utilized. The use of clinical samples allowed for accuracy and precision studies. The workflow involved four main steps; viral RNA extraction, targeted amplicon generation, amplicon sequencing and data analysis. Accuracy of 98% with an average percentage error of 3% was reported for the NGS based assay against the SS platform demonstrating similar performance. The coefficient of variation (CV) findings for both the inter-run and inter-personnel precision showed no variability (CV ≤ 0%) at the relative abundance of ≥ 20%. For both inter-run and inter-personnel, a variation that affected the precision was observed at 1% frequency. Overall, for all the frequencies, CV registered a small range of (0–2%).

**Conclusion:**

The NGS-based in-house HIV genotyping method fulfilled the minimum requirements that support its utilization for drug resistance profiling in a clinical setting of a low-income country. For more inclusive quality control studies, well-characterized wet panels need to be established.

**Supplementary Information:**

The online version contains supplementary material available at 10.1186/s12981-021-00390-8.

## Background

Globally, antiretroviral therapy campaigns have led to a tremendous reduction in morbidity and mortality [1]. Despite this, the risk of virologic failure increases with the emergence and potential transmission of drug-resistant variants which threaten the UNAIDS 95-95-95 goals to control the HIV epidemic worldwide by 2030 [2–4]. Fortunately, interventions such as HIV genotyping that monitor HIV-1 drug resistance (HIVDR) and surveillance of transmitted drug resistance serve a critical role in the fight against HIV/AIDS [5–7].

HIV Genotyping assays are diverse given the dynamic nature of the technique and driven by a vast range of technologies [8]. Currently, commercial assays such as ViroSeq are available in developed countries. However, because of the cost and varying sensitivity across HIV-1 subtypes, these are hardly utilized in resource-limited settings such as Uganda [9, 10]. Instead, most laboratories develop in-house assays that are affordable and designed to genotype HIV-1 subtypes and circulating recombinant forms (CRFs) that are predominant within their localities.

Conventional HIV genotyping using Sanger sequencing techniques serves as the mainstay for clinical HIVDR testing. Although Sanger sequencing has been commonly applied as the “gold standard” for a while, there are some intrinsic limitations with this technology. As opposed to Next Generation Sequencing, the technique has low data throughput and limited capacity to detect variants below 20% intra-host frequency of the quasi-species [11, 12]. Literature suggests that low-abundance HIVDR variants could have a relevant clinical impact and that their detection could benefit treatment management [13–15]. However, a clinically significant threshold is yet to be defined amidst the promising benefits of the new NGS technologies.

Although NGS chemistries differ, all platforms are characterized by high-output, clonal, and parallel sequencing [16]. These outperform conventional Sanger sequencing in scalability, sensitivity, and quantitative detection of minority resistance variants [17]. The sensitive methodology can accurately profile the protease, reverse transcriptase, integrase, and maturation inhibitors, as well as HIV-1 coreceptor tropism in a single run. Despite this, NGS-based assays have been primarily limited to research settings and are rarely used in clinical settings, especially in low- and middle-income countries. This can be attributed to the assay not being standardized, which is necessary for accreditation by regulatory agencies and the lack of an appropriate validation and performance assessment platform for NGS-based HIV genotyping.

Therefore, considering the principles of molecular assay validations and benchmark to the available guiding documents [18], here we present a validation report for the NGS-based assay. The validation report was used to assess the HIVDR assay of the Central Public Health Laboratories (CPHL) which is the national reference laboratory for specialized tests. The report was also used as a benchmark for the NGS assay for Joint Clinical Research Centre (JCRC) which is a WHO-accredited HIVDR laboratory and part of the HIVDR surveillance network.

## Results

### Accuracy

**Fig. 1:**
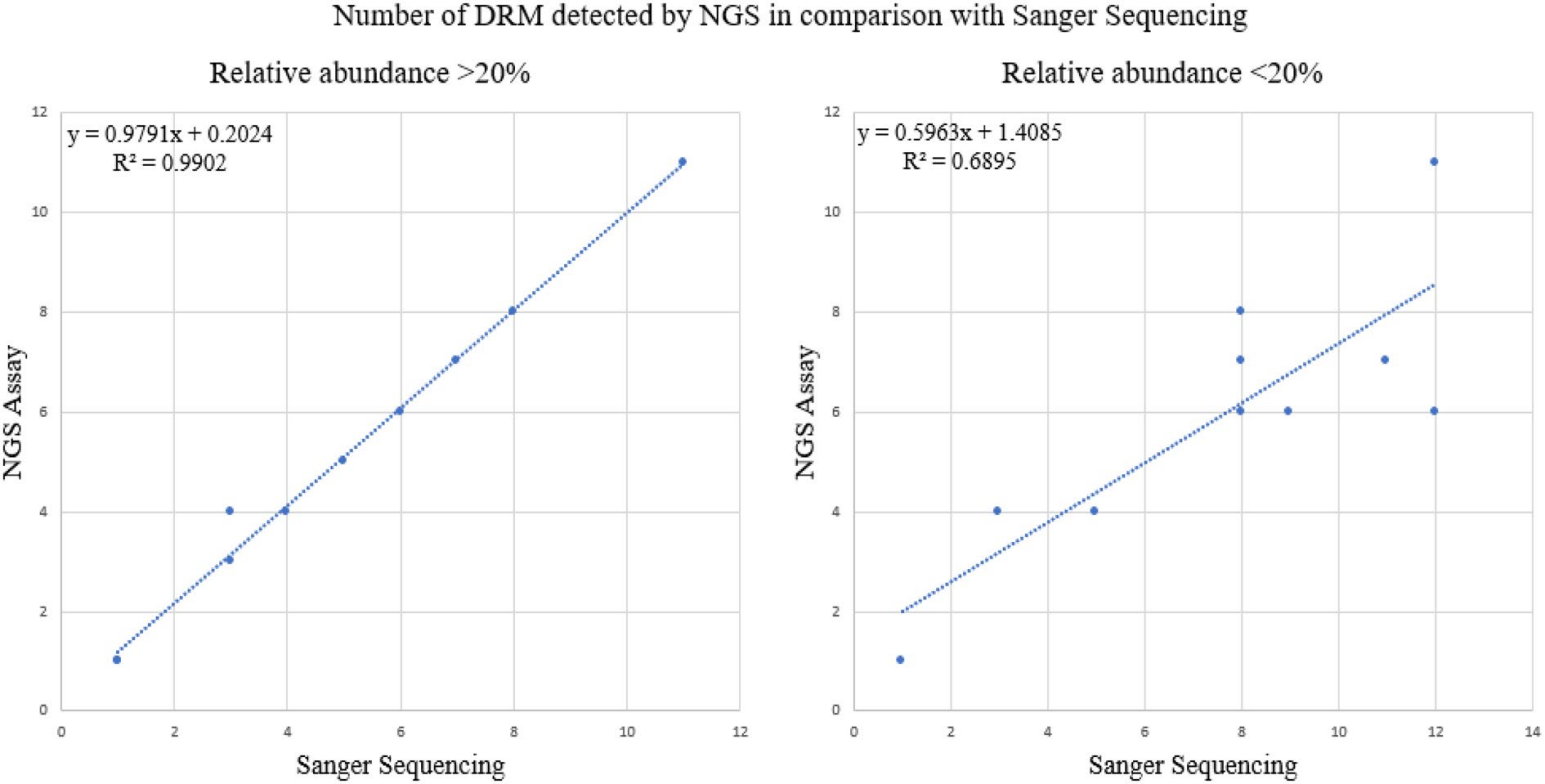
Accuracy demonstrations at relative abundances > 20% and < 20%. Shows correlation between the NGS platform and the gold standard for linear range of mutation detection

Accuracy was demonstrated by sequencing 10 samples at frequencies above 20% which is the threshold for clinical interpretation. Accuracy of 98% with an average percentage error of 3% was reported for the NGS assay against the Sanger sequencing platform demonstrating similar performance, Table 1. Considering the linear range of mutation detection, no significant difference (*r* = 0.99, *p* = 2.5 Pearson coefficient correlation, Fig. 1) was observed between the NGS platform and the gold standard.

**Table 1:** Drug resistance mutation profiles for accuracy of NGS against Sanger Sequencing

Sample ID	Sequencing platform	RT region		PR region	
		NRTI mutations	NNRTI mutations	Major mutations	Accessory
DR-968–19	Sanger Sequencing	K70R, M184V, K219Q	K103N, H221Y, M230L, L234I	None	None
	NGS	K70R, M184V, K219Q, D67G	K103N, H221Y, M230L, L234I	None	None
DR-969–19	Sanger Sequencing	None	K103KNRS	None	None
	NGS	None	K103KNRS	None	None
DR-970–19	Sanger Sequencing	M41L, D67N, M184V, T215F	A98G, G190A	N88S	V32A, L33F
	NGS	M41L, D67N, M184V, T215FIS	A98G, G190A	N88S	V32A, L33F
DR-971–19	Sanger Sequencing	M41L, K65R, M184V, K219N	L100I, K103N	None	None
	NGS	M41L, K65R, M184V, K219N, T215FIS	L100I, K103N, G190A, A98G	N88S	V32A, L33F
DR-972–19	Sanger Sequencing	D67N	K103N, K238T, Y188N	None	None
	NGS	D67GNS	K103N, K238T	None	None
DR-975–19	Sanger Sequencing	M41L, M184V, L210W, T215F	A98G	M46I, I50V, I54V, V82A	L10F, L33F
	NGS	M41L, M184V, L210W, T215F	A98G, V108I	M46I, I50V, I54V, V82A	L10F, L33F
DR-976–19	Sanger Sequencing	M184V	K101E, G190A, E138A	None	None
	NGS	K70R, M184V, K219Q	K101E, G190A, K103N, H221Y, M230L, L234I	None	None
DR-979–19	Sanger Sequencing	T69DN, K70R, M184V	G190S	None	None
	NGS	T69DN, K70R, M184V	G190S	None	G48R
DR-980–19	Sanger Sequencing	M184V, T215Y	K101E, E138A, G190A	None	None
	NGS	M184V, T215NSY	K101E, E138A, G190A, H221Y, P225H	M46I, L76V, I84V	Q58E
DR-981–19	Sanger Sequencing	E44D	None	None	None
	NGS	E44D, K65E, M184IV	A98G, K103N, P225H	I54V, V82A	None

Clinical interpretation of drug resistance mutation profiles is based on frequencies 20% and above. As expected, the linear range of the number of DRMs detection showed a significant reduction in correlation when the data indicated a significant difference (r = 0.7, p < 0.05 Pearson coefficient correlation, Fig. 1) between the two platforms < 20% frequencies. This is attributed to the sequencing depth capacity of the NGS platform as opposed to Sanger sequencing.

### Precision

**Fig. 2:**
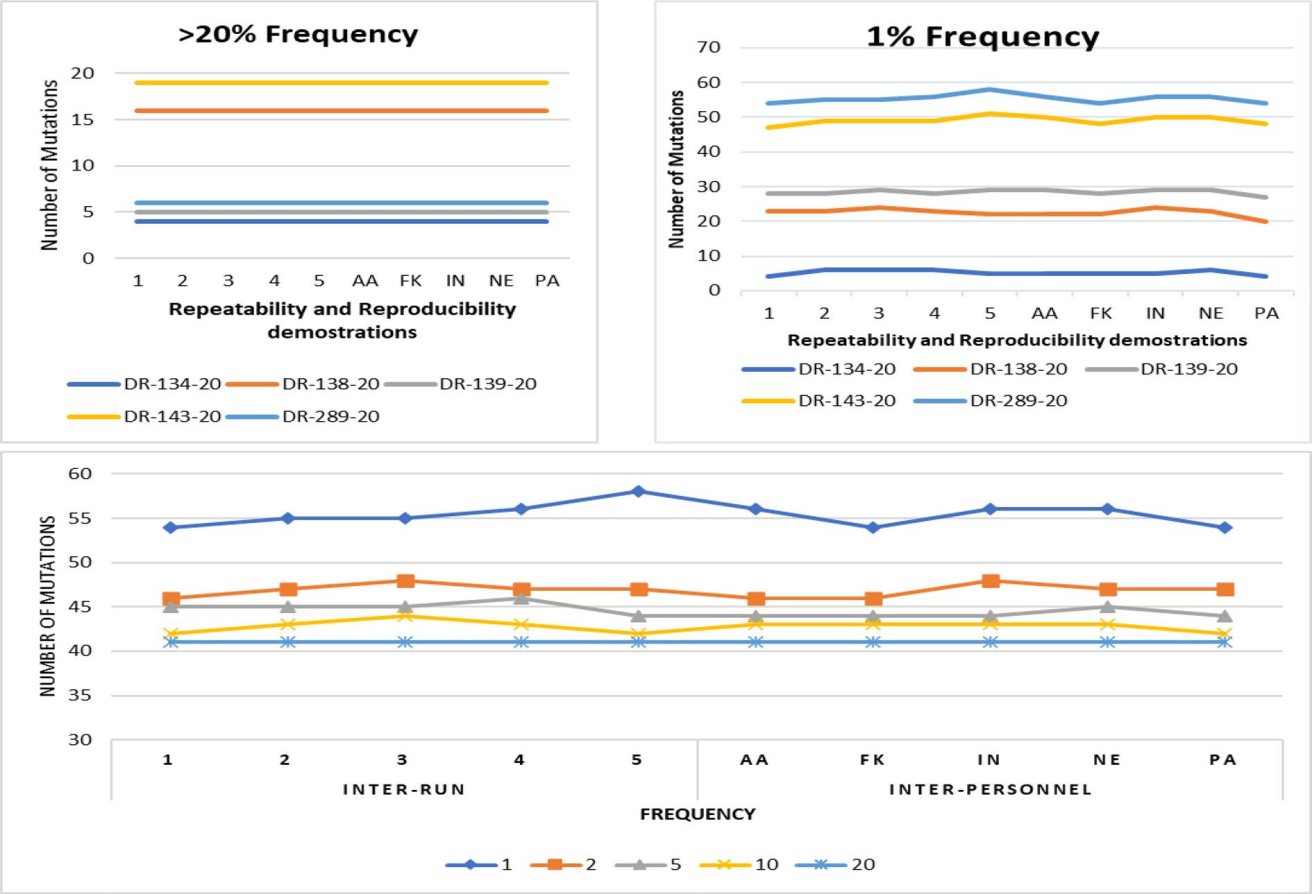
Precision demonstrations for repeatability and reproducibility. Shows the detected mutations at different relative abundances for the inter-run and inter-personnel precision trials

Repeatability (inter-run) was demonstrated by processing and sequencing five samples 5 times using the same conditions in 1 week. Reproducibility (inter-personnel) was demonstrated by processing and sequencing five samples by five laboratory technologists in 1 week. The NGS assay passed the precision demonstrations at frequencies of above 20%, discrepancies occurred at very low frequencies of about 1%. As expected, the NGS assay detected similar mutation profiles at 20% frequency which is the threshold for clinical interpretation of drug resistance mutation profiles, Fig. 2. Despite the difference at a low cut off of 1%, the NGS assay detected comparable similar mutation profiles at frequencies lower than 20%. The difference can be attributed to; inter-personnel and experimental errors i.e., pipetting errors, Fig. 2.

The coefficient of variation (CV) was used to determine the inter-run and inter-personnel precision, Table 2. The findings showed no variability (CV ≤ 0%) at the relative abundance of 20% for both the inter-run and inter-personnel precision. For both inter-run and inter-personnel, the variation that affected the precision was observed at 1% frequency. Overall, for all the frequencies, CV registered a small range of (0%-2%).

**Table 2:** Coefficient of Variation for inter-run and inter-personnel precision

	Coefficient of Variation, CV %				
	DRM Relative abundance (% Frequency)				
Precision	1	2	5	10	20
Inter-Run	2%	1%	1%	2%	0%
Inter-Personnel	2%	2%	1%	1%	0%

**Fig. 3:**
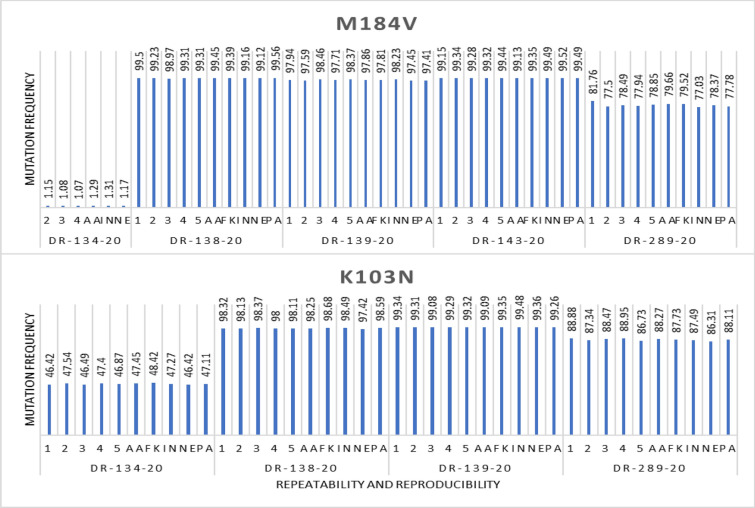
Precision demonstrations for M184V and K103N mutations. Shows the mutation frequencies of common mutations M184V and K103N across the inter-run and inter-personnel demonstrations

Precision demonstrations were further interrogated for the most prevalent mutations that are, M184V and K103N. Figure 3 shows the frequencies of M184V across the precision trials for the five samples. M184V is one of the major and most common mutations among the NRTI class of drugs. M184V causes high-level in vitro and in vivo resistance to lamivudine (3TC) and emtricitabine (FTC) and low-level resistance to Didanosine and Abacavir (ABC). As expected, frequencies > 20% were relatively uniform across all the runs for every sample. However, at frequencies < 2%, M184V was not visible for some runs. Since the frequencies are too low, the difference can be attributed to; inter-personnel and experimental errors i.e., pipetting errors. Figure 3 also shows the frequencies of K103N across the precision runs for four samples. K103N is one of the major and most common mutations among the NNRTI class of drugs. K103N is a non-polymorphic mutation that causes high-level reductions in Nevirapine and Efavirenz susceptibility. As expected, the frequencies > 20% were relatively uniform across all the runs for every sample. The slight variations are negligible and could be attributed to experimental errors. The fifth sample had no K103N mutation in all the demonstrations.

**Fig. 4:**
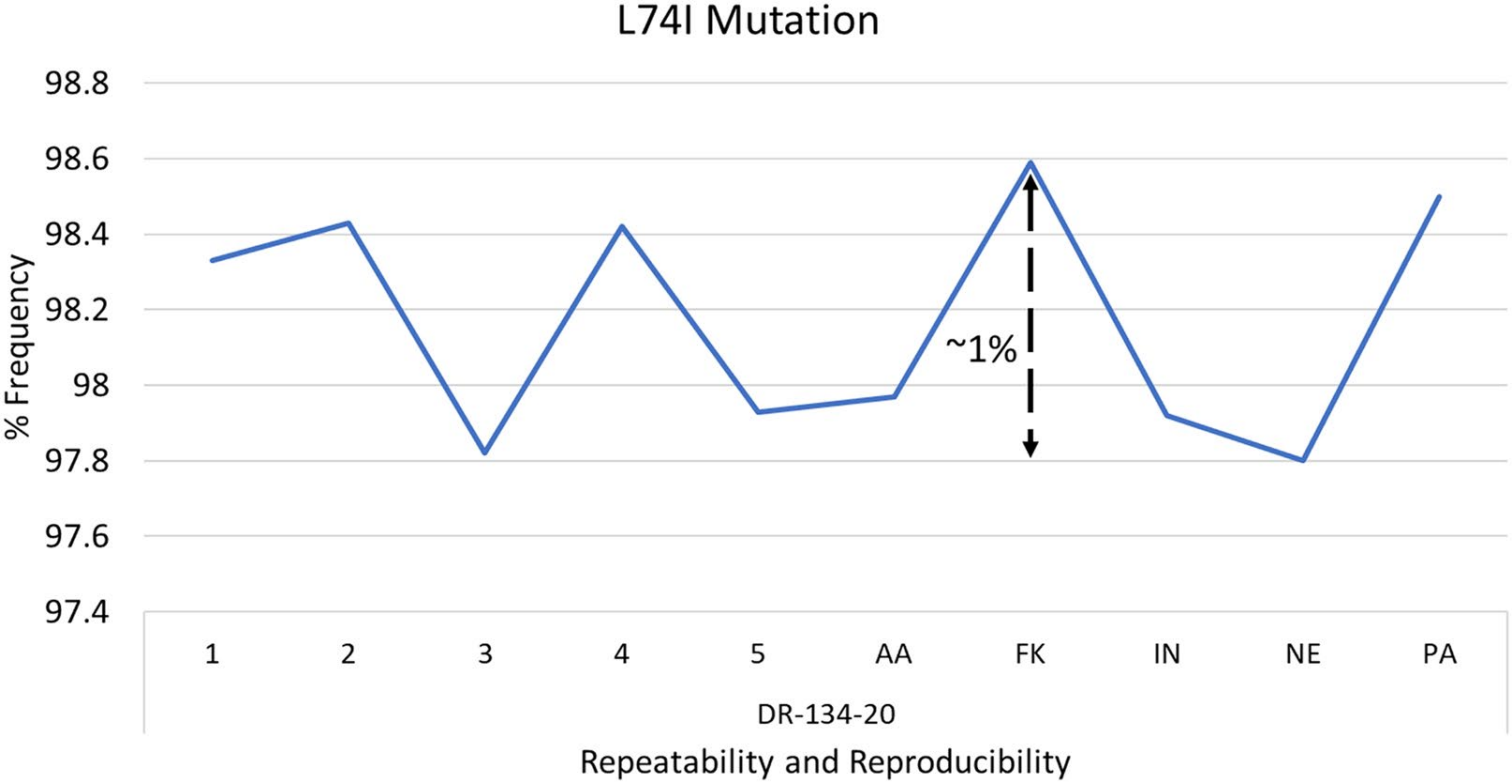
Precision demonstration for L74I mutation. Shows the percentage difference of frequencies across precision trials for the integrase inhibitor mutation L74I

L74I was the only mutation observed for the Integrase Strand Transfer Inhibitors (INSTI). Figure 4 shows the frequencies across the reproducibility and repeatability runs. L74I is a polymorphic accessory mutation commonly selected by each of the INSTI. Although a slight difference of ~ 1% was registered, the assay performed as expected. The difference can be attributed to inter-run and inter-personnel bias.

**Fig. 5:**
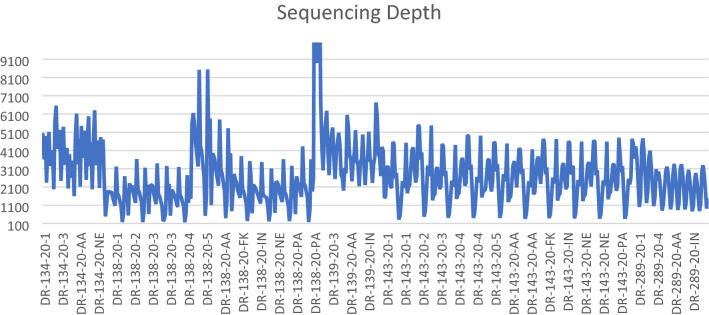
Sequencing depth variations across precision demonstrations. Shows an overview of the sequencing coverage for the precision trials

Overall, the average coverage (sequencing depth) per nucleotide position for precision demonstrations was 2795 as also illustrated in Fig. 5.

## Discussion

HIV genotyping is increasingly picking pace in Uganda despite resource limitations. This highly specialized molecular test has been a preserve for research purposes for decades which is attributed to operational costs. Mobilization of resources for public health comes at a time when HIV genotyping is advancing technologically. The conventional technology that is currently utilized in the country is based on the Sanger sequencing method. The method is faced with limitations such as low data output and the inability to detect mutations of the viral minority populations. Despite this, SS still serves as a gold standard primarily because the NGS technologies lack a standardized validation strategy and a consensus is yet to be reached on the relevance of the rich data output.

Nevertheless, CPHL acquired an NGS platform for use in clinical diagnostics. Therefore, in this report, we present a validation report of the in-house assay based on NGS technologies. The assay was validated on two criteria; accuracy and precision. Accuracy of 98% was reported for the NGS assay against the SS assay. The calculations were set at 20% as the mutation frequency threshold given that SS assays can arguably detect mutations in approximately 15% of the viral population variants. Qualitatively all mutations reported on SS were also reported on the NGS platform, Table 1. This was expected as NGS is documented to have a higher sequencing depth as compared to SS. The assay can massively generate data without compromising on quality. A significant difference was observed when the threshold was set below 20%, the findings actualize the literature that the NGS platforms can detect mutations occurring at low frequencies as opposed to the SS platforms [17]. Although the NGS platform performed as expected against SS, this approach oversimplifies the complexity and richness of NGS HIVDR data that can report on a highly diverse population of HIV. Therefore, well-validated External Quality Assurance (EQA) strategies that check the NGS platform remain to be established.

The coefficient of variation was used to study precision. The report documented no variation (CV ≤ 0%) at the relative abundance of 20%. However, slight variations that affected precision were noted at relative abundances below 20%, but more prominent at 1%. This could be attributed to the sequence error rate of ~ 1% and the increased likelihood of cross-contamination and sampling/PCR biases [19]. Although, minority mutations at frequencies < 0.5% could result in treatment failure [20], the clinical relevance of variants below 5% is still a question of debate [21]. However, if the variants below 5% are proven relevant, the validation process and quality assurance strategies could have to leverage the use of unique molecular identifiers (UMIs) to determine the accuracy of NGS assays at such lower frequencies [22].

We acknowledge the limitation of using clinical samples as opposed to well-characterized wet panels. Because of this, Sensitivity, Specificity and the Lower limit of detection (LOD) were not studied. Sensitivity determines the probability of the assay to detect mutations when present. Specificity determines the probability of the assay not detecting mutations when absent. LOD determines the lowest viral load at which the assay can still be able to detect all the mutations present. The true picture of the above-mentioned parameters could not be achieved with clinical samples consisting of viral RNA of variable quality, quantity and variant diversity. However, even for accuracy and precision, a possibility for bias could have been introduced due to the error-prone reverse transcription PCR step and sequencing error [23]

## Conclusion

The NGS assay presents opportunities to revolutionize the field of HIV Genotyping. However, these are under-exploited partly because, their relevance has not been well studied in the context of clinical management. This has left such a powerful tool to be oversimplified when compared with inferior assays such as SS. In this report, we validated the NGS assay against the SS assay. The NGS assay proved fit for HIVDR testing in a clinical setting of a low resource country. However, with the advance in research, a well elaborate quality control strategy needs to be established.

## Methods

**Fig. 6:**
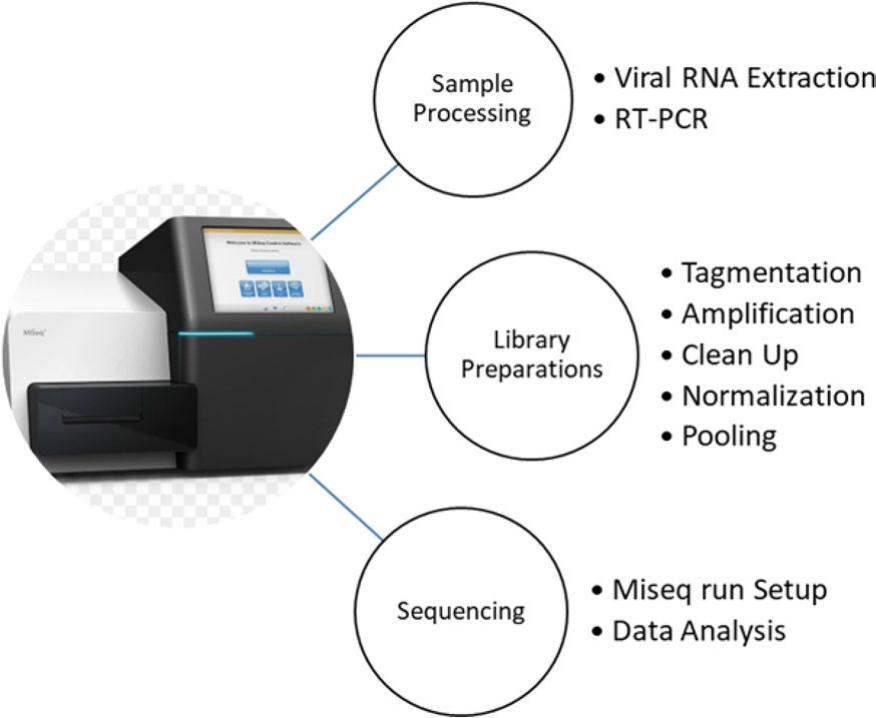
Pictorial view of HIV genotyping processes. Shows the experimental steps for NGS platform from viral RNA extraction to analysis of data

In the absence of established proficiency test panels for NGS-based HIVDR testing, 15 clinical plasma samples for routine care were utilized as shown in Table 3. These were collected for routine HIV-1 drug resistance testing as the standard of care for HIV-infected individuals failing treatment. The selected samples and size represented the predominantly circulating variants of HIV-1 in Uganda, this was sufficient for accuracy and precision demonstrations. However, the size was also pre-determined by the availability of resources. Briefly in Fig. 6, Viral RNA was extracted using Qiagen QIAmp Viral RNA kit [24].

**Table 3:** Experimental sample Characteristics

Sample ID	HIV-1 subtypes	Demonstrations	Sequenced Genes
DR-968-19	D	Accuracy	RT, PR
DR-969-19	D	Accuracy	RT, PR
DR-970-19	A	Accuracy	RT, PR
DR-971-19	D	Accuracy	RT, PR
DR-972-19	A	Accuracy	RT, PR
DR-975-19	A	Accuracy	RT, PR
DR-976-19	B	Accuracy	RT, PR
DR-979-19	B	Accuracy	RT, PR
DR-980-19	D	Accuracy	RT, PR
DR-981-19	C	Accuracy	RT, PR
DR-134-20	A	Precision	RT, PR, INT
DR-138-20	C	Precision	RT, PR, INT
DR-139-20	D	Precision	RT, PR, INT
DR-143-20	D	Precision	RT, PR, INT
DR-289-20	A	Precision	RT, PR, INT

RT-PCR was done using a Superscript III One-Step system with Platinum Taq [25]. Amplification of the target genes in the pol region was done using two (2) pairs of external and nested primers; External primers (Pair 1 covering the protease gene and reverse transcriptase spanning between positions 2058 and 3529; (forward, GAAAGACTGCACTGAAAGACAGGC), (reverse, GCTATTAAGTCTTTTGATGGGTCAT), Pair 2 covering the integrase gene spanning between positions 3694 and 5455; (forward, TATGGGGAAAGACTCCTAAATTTA), (reverse, GTCCTGCTTGATATTCACACC)). Nested primers (Pair 1 covering the protease gene and reverse transcriptase spanning between positions 2155 and 3323; (forward, ACAGCCCCACCAGCAGAG), (reverse, CTGTATATCATTGACAGTCCAGCT), Pair 2 covering the integrase gene spanning between positions 4022 and 5258; (forward, AGAAGTAAACATAGTAACAGACTCACA) (reverse, TGCAGACCCCAATATGTTCTA)) [26]. The primer set allows for the robustness of the assay with the ability to genotype all circulating HIV-1 subtypes in the country, Table 3.

Amplicons generated using the aforementioned nested primers were used to prepare NGS libraries using the Nextera XT DNA library preparation kit [27]. The amplicons for the protease and reverse transcriptase region were generated separately from the integrase region, however, these were combined before library preparation at proportional concentrations for sequencing in the same pooled run. Each pooled library had 25 samples; these were run using the Miseq V2 reagent kit on the Miseq system [28] at CPHL. Sanger sequencing was done using BigDye Terminator v3.1 cycle sequencing kit [29] on the ABI genetic analyzer 3730xl [30] at JCRC. To achieve double coverage, two sets were used for forward and reverse primers, in addition to the aforementioned nested primers, the following were also added; for protease and reverse transcriptase region: forward (CTGTACCAGTAAAATTAAAGCCAGG), reverse (TCTTCTGTCAATGGCCATTGTTTA), for integrase region: reverse (TGCAGACCCCAATATGTTCTA). Base-calling parameters were set to end at PCR stop, the quality threshold was set to assign Ns to bases with quality values (QVs) less than 15, peak heights were also considered to reduce background noise.

### Data analysis

Data generated was in two formats; FASTA and FASTQ depending on the sequencing platform. FASTA formats are created from chromatograms generated by the Sanger sequencing platforms. Raw chromatograms were analyzed using a web-based software RECall as recommended by World Health Organization (WHO) for laboratories in the HIVDR network [31]. The RECall is an automated base-calling software [32]. This enabled us to do quality checks on; raw chromatogram quality, single-stranded coverage, sequence length, stop codon, excessive mixtures, bad insertions, ambiguous nucleotide/amino acids, APOBEC mutations, atypical mutations and genetic distance. Among the outputs of the RECall analysis included; a consensus sequence, an excel file with the mutation list, a CSV file with the susceptibility scores and pdf files including an image of a neighbour-joining tree.

FASTQ formats were generated from the NGS platforms. These are text-based formats for nucleotide sequences and their corresponding quality scores. Analysis of the FASTQ files was done using an online-based pipeline, Hydra [33]. Among the outputs from Hydra included; a consensus sequence and an Amino Acid Variant Format (AAVF) file. The AAVF file report provides a compact summary of the amino acid variation obtained by conceptual translation of the NGS read pileup across the examined region of the HIV genome [34]. The AAVF files for the samples were then uploaded to the Stanford University HIV Drug Resistance Database [35] for drug resistance profiling.

### Criteria for assessment of the HIVDR NGS based home-brewed assay

Accuracy is defined as the closeness of the measurements to the gold standard. Accuracy was demonstrated by sequencing 10 samples on both platforms. For each sample, DRMs detected by SS were compared with those detected by NGS and their number was plotted on scatter graphs. Accuracy was calculated in percentages by comparing the number of DRMs detected by NGS with the standard SS. An average percentage for the 10 samples was established as the accuracy. Precision is defined as how close the agreement of the outcomes is between repeated measurements. This was categorized into Repeatability -the variation arising when all efforts are made to keep conditions constant by using the same instrument and operator and repeating the measurements during a short period. Reproducibility -the variation arising using the same measurement process among different instruments or operators over longer periods. Repeatability was demonstrated by processing and sequencing five samples 5 times using the same conditions in 1 week. Reproducibility was demonstrated by processing and sequencing five samples by five laboratory personnel in 1 week designated by the initials AA, FK, IN, NE, PA.

## Supplementary Information

**Additional file 1.** Table showing the detailed precision report from the Miseq system.

## Data Availability

All data generated or analyzed during this study are included in this published article [and its Additional file 1].

## Abbreviations

APOBEC: Apolipoprotein B mRNA editing enzyme, catalytic polypeptide-like gene
AAVF: Amino Acid Variant Format
NGS: Next Generation Sequencing
SS: Sanger Sequencing
UNHLS: Uganda National Health Laboratory Services
JCRC: Joint Clinical Research Center
PCR: Polymerase Chain Reaction
NNRTI: Non-nucleoside reverse transcriptase Inhibitors
EQA: External Quality Assessment
INSTI: Integrase Strand Transfer Inhibitors
DRM: Drug Resistance Mutations
HIVDR: HIV drug resistance
